# KEY LEARNING FOR TWELVE UNECOM MEDICAL STUDENTS IMMERSED FOR 48 HOURS IN AN ACUTE CARE HOSPICE HOME DURING 2017-18

**DOI:** 10.1093/geroni/igz038.3124

**Published:** 2019-11-08

**Authors:** Emily Tamimie, Marilyn R Gugliucci

**Affiliations:** University of New England College of Osteopathic Medicine, Biddeford, Maine, United States

## Abstract

Introduction: Most US medical schools are not able to provide practical experiences in end-of-life or palliative care. The University of New England College of Osteopathic Medicine Learning by Living 48 Hour Hospice Home Immersion (HHI) Project provides intense learning for second through fourth year medical students. Students are immersed into an acute care 18-bed in-patient hospice home for 48 hours to provide patient care, family support, and post-mortem. Students work with an Interprofessional staff team and independently. Methods: The HHI utilizes qualitative ethnographic/autobiographic research designs. Two key research questions include: (1) What is it like for ME to live in the Hospice Home for 48 hours? and (2) What will I take from this experience to my future as a practitioner?” Each student writes a journal during the three research stages; pre-fieldwork; fieldwork; post-fieldwork. Twelve 2nd year medical student journals randomly selected from 2017-18 immersions (N=24) were analyzed 5/2019-7/2019. Detailed qualitative manual and content analysis utilizing established interrater-reliability procedures were conducted on 300+ pages of data (UNECOM Morgane Student Research Fellowship). Results: Of the many themes identified, three key themes were notable for all 12 students for question one: Religion/Spirituality; Acceptance; and Reactions to Death. Key takeaways for question two included being: (a) able to have conversations about death; (b) at peace with death; (c) present with death, and knowing death is a part of life. Conclusion: Each student experienced his/her immersion differently, but all expressed this was a life-altering project providing critical education on hospice and end-of life care.

